# Tuning Ciprofloxacin Release Profiles from Liposomally Encapsulated Nanocrystalline Drug

**DOI:** 10.1007/s11095-016-2002-5

**Published:** 2016-07-20

**Authors:** David Cipolla, Huiying Wu, Simon Eastman, Tom Redelmeier, Igor Gonda, Hak-Kim Chan

**Affiliations:** 1 Advanced Drug Delivery Group, Faculty of Pharmacy, The University of Sydney, Pharmacy Building A15, Sydney, NSW 2006 Australia; 2 Aradigm Corporation, 3929 Point Eden Way, Hayward, California 94545 USA; 3 ProNAi Therapeutics, Inc., 2150 - 885 W. Georgia St., Vancouver, British Columbia V6C 3G1 Canada; 4 Northern Lipids Inc, 8855 Northbrook Court, Burnaby, British Columbia Canada V5J 5J1

**Keywords:** *in vitro* release, liposome, nanocrystal, personalized medicine, surfactant

## Abstract

**Purpose:**

In order to attenuate the drug release rate, a single freeze-thaw step was previously shown to convert encapsulated drug into a single nanocrystal within each liposome vesicle. The goal of this study was to alter the nanocrystalline character, and thus the drug encapsulation state and release profile, by addition of surfactant prior to freeze-thaw.

**Methods:**

A liposomal ciprofloxacin (CFI) formulation was modified by the addition of surfactant and frozen. After thawing, these formulations were characterized in terms of drug encapsulation by centrifugation-filtration, liposome structure by cryo-TEM imaging, vesicle size by dynamic light scattering, and *in vitro* release (IVR) performance.

**Results:**

The addition of increasing levels of polysorbate 20 (0.05 to 0.4%) or Brij 30 (0.05 to 0.3%) to the CFI preparations followed by subsequent freeze-thaw, resulted in a greater proportion of vesicles without drug nanocrystals and reduced the extent of growth of the nanocrystals thus leading to modified release rates including an increase in the ratio of non-encapsulated to sustained release of drug.

**Conclusions:**

This study provides another lever to achieve the desired release rate profile from a liposomal formulation by addition of surfactant and subsequent freeze-thaw, and thus may provide a personalized approach to treating patients.

## Introduction

Liposomes are lipid vesicles, typically composed of bilayers of phospholipids and sterols. In aqueous media, the two hydrophobic fatty acid tails of each phospholipid molecule are oriented towards the center of the bilayer while the hydrophilic head group is oriented towards the internal or external aqueous phase. Hydrophilic drugs can be loaded into the aqueous interior of the vesicles while lipid soluble drugs can be associated with the lipid bilayer. A number of pharmaceutical products are now approved using liposomal formulations, and many more are in late-stages of clinical trials. Liposomal products have been designed to improve the safety and efficacy of treatment, through modification of the pharmacokinetics and biodistribution of the associated API (1, 2). Liposomes can also incorporate targeting ligands, polyethylene glycol to increase the circulation lifetime, or be further modified to trigger drug release in response to external stimuli (*e.g.*, heat, light or ultrasound) or local stimuli (*e.g.*, changes in pH) (1–3). Thus, liposomes represent a versatile class of drug carriers which are continuing to evolve to address new therapeutic opportunities. These studies describe another step in the liposome evolution process by expanding the formulation toolbox to modulate the drug release profile with only relatively minor changes in composition of the formulation. In particular, the key impact appears to be the modification of the solid state of the encapsulated drug, in this example ciprofloxacin.

A liposomal ciprofloxacin formulation (CFI) composed of 80 to 90 nm unilamellar vesicles was used as the model formulation in these studies (4). CFI containing >99% encapsulated ciprofloxacin, termed Lipoquin® (Aradigm, Inc., Hayward, CA), and a formulation of 70% encapsulated ciprofloxacin and 30% non-encapsulated ciprofloxacin, termed Pulmaquin® (Aradigm, Inc., Hayward, CA), have shown promise in treating lung infections *via* inhalation delivery in cystic fibrosis (CF) (4, 5) and non-CF bronchiectasis (NCFB) (4, 6). Because unencapsulated ciprofloxacin is poorly soluble in CFI at pH 6.0, Pulmaquin consists of a two-vial preparation of CFI (liposomally-encapsulated ciprofloxacin) and non-encapsulated ciprofloxacin (4). Pulmaquin has advanced into late stages of clinical trials in NCFB (4). These formulations have also shown efficacy in a number of biodefense applications including *Yersinia pestis* (4, 7), *Francisella tularensis* (4, 8), and Q fever (4, 9). However, lung infections can differ in a number of key attributes including the location of the infection in the lungs (intracellular or extracellular), the presence of biofilm and the sensitivity of the infectious agent to antibiotics. Additionally, some antibiotics are ‘time-dependent’ and are more effective the longer their concentration remains above the bacteria’s minimum inhibitory concentration (MIC), while others are ‘concentration-dependent’ and are more effective the higher the peak concentration is above the MIC; *e.g.*, aminoglycosides, while still others, like fluoroquinolones, may require a blending of these two elements (4, 10). Thus a combination of a bolus-release and sustained release profile may be ideal for ciprofloxacin as was designed into the Pulmaquin formulation. Depending on the specific infection and site of disease, modifications to both the ratio of the bolus to the sustained release component, and to the timing and duration of the sustained release component itself, may be desirable to improve efficacy.

The rate of drug release from liposomes can be modulated by judicious choice of the phospholipid and the ratio of lipid to sterol. Other factors can also affect the release profile including the vesicle size distribution, the properties of the drug, the method of drug loading into the liposomes, the location of the drug within the vesicles, and ultimately the route of administration and the resulting biological fluids that come into contact with the liposomes (1, 2). While it is not typically possible to modify the drug release rate once the liposome composition has been defined and the formulation has been manufactured, recently it was demonstrated for a liposomal ciprofloxacin formulation that the drug release rate could be increased by addition of polysorbate 20 (0.2 to 0.4%) or polysorbate 80 (0.2%) under conditions of osmotic swelling (11). Alternatively, the release rate for the original liposomal ciprofloxacin formulation could be decreased by conversion of the encapsulated ciprofloxacin into nanocrystals following a single freeze-thaw step (12). These two advances create options for faster or slower releasing formulations and could be used to personalize therapy so that the release profile optimally targets the infectious agent, with minimum changes in the qualitative composition or manufacturing processes.

The objective of these studies was to further investigate the ciprofloxacin nanocrystal formation processes within the liposomal formulation with the aim to identify procedures that could be used to alter the size or shape of the crystalline drug in a controlled manner. The formation of drug nanocrystals results in lower soluble drug concentrations within the liposomes which would be expected to reduce the rate of drug release across the lipid membrane barrier as has previously been shown for liposomal formulations of precipitated vincristine (13) and doxorubicin (14). The mechanistic model for release of precipitated doxorubicin sulfate from the liposomes predicts that dissolution of the precipitated drug is not rate-limiting; in other words, the driving force for release is the soluble intra-vesicular drug concentration which remains constant if the rate of drug dissolution matches the release of drug from the liposomes (14). Forming ciprofloxacin nanocrystals with different sizes or shapes, as will be attempted in these studies, may provide evidence of whether drug dissolution is rate-limiting for this liposomal system.

As noted above, the addition of nonionic surfactant to a liposomal ciprofloxacin formulation caused transient drug leakage from the vesicles in a concentration-dependent manner but also affected the membrane permeability resulting in faster releasing vesicles (11). Thus, one method to further modulate the release profile of the nanocrystalline liposome formulations may be the addition of nonionic surfactant prior to freeze-thaw. The presence of surfactant may result in a reduction in the amount of encapsulated drug; a smaller internal reservoir of drug may lead to smaller drug crystals. If the dissolution step is rate-limiting, then smaller drug crystals may result in a relatively faster release profile than for larger drug crystals. In contrast, if the dissolution step is not rate-limiting, then the change in crystal structure may have no effect on the rate of drug release from the nanocrystalline component. The addition of surfactant followed by freeze-thaw may provide additional levers to adjust the ratio of the drug bolus to the sustained release profile of a nanocrystalline liposomal ciprofloxacin formulation.

## Materials and Methods

### Materials

Liposomes containing 50 mg/ml ciprofloxacin (expressed in terms of ciprofloxacin hydrochloride) in a pH 6.0 histidine buffer were manufactured by Northern Lipids Inc. (Burnaby, BC, Canada) and Sigma-Tau (Indianapolis, IN). The following materials were used for the preparation, characterization or analysis of the liposomal ciprofloxacin (CFI) formulations: sucrose (Sigma-Aldrich (St. Louis, MO)), HEPES, free acid (Avantor (Center Valley, PA)), sodium chloride (Amresco (Solon, OH)), HPLC grade methanol (Fisher Scientific (Fair Lawn, NJ)), triethylamine (TEA, JT Baker (USA)), polysorbate 20 (VWR Int. (West Chester, NJ)), polyethylene glycol dodecyl ether (Brij 30 (Sigma-Aldrich (Australia))), Donor Adult Bovine Serum (HyClone (Logan, Utah)), and Nanosep centrifugal filtration devices, 10 K and 30 K molecular weight (Pall Corporation (Ann Arbor, MI)). Deionized water was used for all studies.

### Methods

#### Liposomal Ciprofloxacin Preparation

The preparation of CFI, an aqueous dispersion of ~80 nm liposomes containing cholesterol and hydrogenated soy phosphatidylcholine (HSPC), has been reported previously (4, 11, 15). Briefly, multilamellar liposomes were extruded through membranes to produce predominantly 80 to 90 nm sized unilamellar liposomes that were subsequently actively loaded with ciprofloxacin (4, 16, 17). Any unencapsulated ciprofloxacin was removed by diafiltration resulting in >99% encapsulated ciprofloxacin at a target concentration of 50 mg/ml.

#### Freeze-Thaw Studies to Create Nanocrystals

Formulations of 50 mg/ml CFI were diluted with various combinations of sucrose, trehalose, polysorbate 20, Brij 30 or water to produce liposomal formulations targeting a ciprofloxacin concentration of 12.5 mg/ml. One ml aliquots were transferred to either plastic Eppendorf tubes or HPLC glass vials and frozen in either liquid nitrogen or by storing for at least 20 h in a −50°C freezer. The samples were thawed at room temperature and vortexed to ensure homogeneity prior to subsequent evaluation of physical appearance, drug encapsulation, vesicle size, *in vitro* release, or cryo-TEM analysis. Following thawing, stability to freeze-thaw was confirmed by visual clarity. The presence of precipitated matter, representing agglomerated vesicles, indicated that the formulation was not stable to freeze-thaw.

#### Vesicle Size

The vesicle size of each sample was measured using a Submicron Particle Sizer Autodilute Model 370 (Nicomp, USA). The measurements were made using the following settings: run time: 5 min; viscosity: 0.933; refractive index: 1.333; temperature: 23°C; scattering angle: 90; intensity set point: 300 KHz; channel width: 10 μsec; mode: vesicle; Gaussian distribution. Each CFI sample was diluted with saline to a concentration of ~2 mg/ml liposomes (1 mg/ml ciprofloxacin), and 0.5 ml was pipetted into a disposable culture tube (Kimble Glass Inc., USA) for analysis. The mean vesicle size and standard deviation (SD) of the distribution are recorded.

#### Drug Encapsulation

Centrifugal filtration was performed using Nanosep Omega centrifugation devices (Pall Corporation, Ann Arbor, MI) with modified polyethersulfone membrane filters of 10,000 or 30,000 molecular weight cut-offs to separate liposomal encapsulated drug from non-encapsulated drug (18). Briefly, each sample was diluted with acetate buffer (50 mM sodium acetate, 145 mM NaCl, pH 4.0) to ~0.625 mg/ml ciprofloxacin and 400 μl was transferred to the centrifugation device and centrifuged for 10 min at 10,000 rpm (8,100 g). The non-encapsulated drug was recovered in the filtrate and quantified by HPLC. The total amount of ciprofloxacin was also determined by HPLC by diluting the original CFI sample twenty-fold into 80% methanol to solubilize the liposomes. The percent drug encapsulation in each sample was calculated by dividing the non-encapsulated ciprofloxacin by the total ciprofloxacin and multiplying by 100%.

#### *In Vitro* Release (IVR) Assay

The rate and extent of release of ciprofloxacin from liposomes was measured in an IVR assay (18). Briefly, the CFI samples were diluted with HEPES Buffered Saline (HBS: 20 mM HEPES, 145 mM NaCl, pH 7.4) to a ciprofloxacin concentration of 50 μg/ml, mixed with chilled (2–8°C) bovine serum (Hyclone) to a ciprofloxacin concentration of 25 μg/ml and placed in a shaking water bath (Techne, TSBS40 (Staffordshire, UK)) at 37°C and 150 rpm. Samples in duplicate were removed after incubation for 30, 60, 120 and 240 min, diluted to a ciprofloxacin concentration of 12.5 μg/ml with chilled (2–8°C) HBS and immersed in an ice-water bath to terminate any further release of encapsulated drug. The amount of non-encapsulated (released) and total drug was measured by centrifugal filtration as described in the drug encapsulation methodology section with one adjustment: The presence of the serum in the CFI sample during centrifugal filtration reduces the recovery of non-encapsulated drug from 100 to 93% so the non-encapsulated drug value is normalized by dividing by 0.93 (18). Due to the presence of substantial unencapsulated drug in some formulations at T_0min_, the normalized initial release rate was calculated by measuring the initial release, T_30min_-T_0min_, divided by the total possible release, 100-T_0min_, and converting to a percentage: 100*(T_30_-T_0_)/(100-T_0_).

#### Cryogenic Transmission Electron Microscopy (Cryo-TEM)

To obtain visual images of the liposomal ciprofloxacin formulations and confirm the presence or absence of drug nanocrystals, and the state of liposome morphology, cryo-TEM analysis was performed either prior to freezing (control) or after freeze-thaw (experimental) using a JEOL 2100 (Tokyo, Japan) instrument operated at 200 kV. In one set of studies, the kinetics of nanocrystal formation were evaluated by performing cryo-TEM analysis immediately after thawing, ~45 min, ~2 h or up to ~16 h after thawing. The control and the thawed CFI samples were diluted to ~5 mg/ml (10 mg/ml liposomes) with water and 3 μl of each sample was applied to a glow discharge Quantifoil carbon grid (Jena, Germany) in a chamber controlled to 22°C and 100% RH. Grids were blotted once with filter paper, at a blotting angle of 2 mm for 2 s, and vitrified by plunging into liquid ethane using a Vitrobot (F.E.I., Eindhoven, Netherlands). The vitrified samples were stored in liquid nitrogen prior to cryo-TEM analysis.

#### High Performance Liquid Chromatography (HPLC) Assay of Ciprofloxacin

An isocratic reverse phase HPLC method operated at a flow rate of 0.9 ml/min was used to quantify the amount of ciprofloxacin in each sample as described previously (11, 18). The mobile phase was a mixture of 0.5% TEA in water, pH 3.0 and 100% methanol (83:17 v/v). Separation was performed using a Nucleosil C-18 column (5 μm, 4.6 × 150 mm, Canadian Life Science, CA) protected with a Nucleosil C-18 guard column (4 × 3.0 mm, Phenomenex, USA) both equilibrated at 35°C. Ciprofloxacin was quantified at 277 nm.

#### Statistical Analysis

The *in vitro* release profiles were compared using difference factor (f_1_) and similarity factor (f_2_) analysis. Similarity factor analysis is recommended by the FDA for modified release solid oral dosage forms (19), with f_2_ values greater than 50 (50 to 100) indicating similarity. Likewise, difference factor values (f_1_) less than 15 (0 to 15) indicate no difference (20). Only the similarity factors are reported here because there was complete agreement between the similarity factor analysis and the difference factor analysis. That is, in every instance that the f_2_ values indicated similarity (f_2_ > 50), the f_1_ values indicated no difference (f_1_ < 15), and in every instance when the f_2_ values indicated lack of similarity (f_2_ < 50), the f_1_ values indicated a difference (f_1_ > 15).

## Results

### Effect of Addition of Surfactant to CFI or Empty Liposomes

Previously we demonstrated formation of drug nanocrystals following freeze thaw for three liposomal ciprofloxacin (CFI) formulations each containing sucrose external to the vesicles, but varying in their internal sucrose concentrations (0, 50 or 150 mM sucrose) (12). The presence of the drug nanocrystals led to a reduction in the *in vitro* release rate (12). In these experiments the goal was to evaluate the addition of surfactant to the CFI formulation to determine if drug nanocrystals would still form after freeze-thaw, and whether the presence of the surfactant would result in modified properties due to interactions of the surfactant with the lipid membrane or a modification to the nanocrystal character.

CFI formulations at 12.5 mg/ml were prepared containing 0.05, 0.1, 0.15, 0.2, 0.25, 0.3, 0.35 or 0.4% polysorbate 20, and 45, 67.5 or 90 mg/ml sucrose to result in a ratio of cryoprotectant to lipid of 2:1, 3:1 and 4:1 (w/w), respectively. CFI formulations at 12.5 mg/mL were also prepared containing 0.05, 0.1, 0.2, and 0.3% Brij 30 and 90 mg/ml sucrose to result in a ratio of cryoprotectant to lipid of 4:1 (w/w). Samples for each of these conditions were evaluated for physical appearance, vesicle size and drug encapsulation pre and post freeze-thaw. A subset of the CFI samples at 12.5 mg/ml containing polysorbate 20 or Brij 30 was then evaluated for IVR and cryo-TEM analysis.

A number of additional surfactants were tested including polysorbate 80, sorbitan monolaurate (SPAN 20), sorbitan monooleate (SPAN 80), poloxamer L44 and poloxamer L62, without success: CFI formulations containing sucrose and these surfactants were unstable to the freeze-thaw process, resulting in agglomerated lipid deposits in the vials.

### Physical Appearance Pre and Post Freeze-Thaw

Prior to freeze-thaw, each of the 28 samples at 12.5 mg/ml CFI containing polysorbate 20 (*n* = 24) or Brij 30 (*n* = 4) retained an appearance similar to that for CFI diluted in sucrose alone; there was no precipitated material after the addition of surfactant. Immediately after freeze-thaw, all of the samples retained clarity indicating stability to freeze-thaw; however, the samples with only 2:1 (w/w) sucrose to lipid started to become turbid over the subsequent 30 min, suggesting inadequate physical stability post freeze-thaw. The presence of surfactant, at least in the samples with adequate cryoprotectant (≥3:1 sucrose to lipid (w/w)), did not appear to compromise the stability of the liposomes in response to freeze-thaw. For the samples with 3:1 and 4:1 (w/w) sucrose to lipid, while they were physically stable to freeze-thaw, a different instability phenomenon was observed over the subsequent 24 h. The samples became birefringent under light microscope displaying the presence of long drug crystals external to the liposomes. Presumably, encapsulated drug was released from the liposomes post freeze-thaw resulting in an external drug concentration that exceeded the solubility limit in the pH 6.0 buffer which led to drug crystallization outside the liposomes (21). The CFI samples containing 0.05, 0.10, 0.15 and 0.20% polysorbate 20 and all CFI samples containing Brij 30 retained clarity for the longest period post freeze-thaw suggesting that they may have lost the least amount of encapsulated drug.

### Vesicle Size Before and After Freeze-Thaw

#### CFI Containing Polysorbate 20

**Fig. 1:**
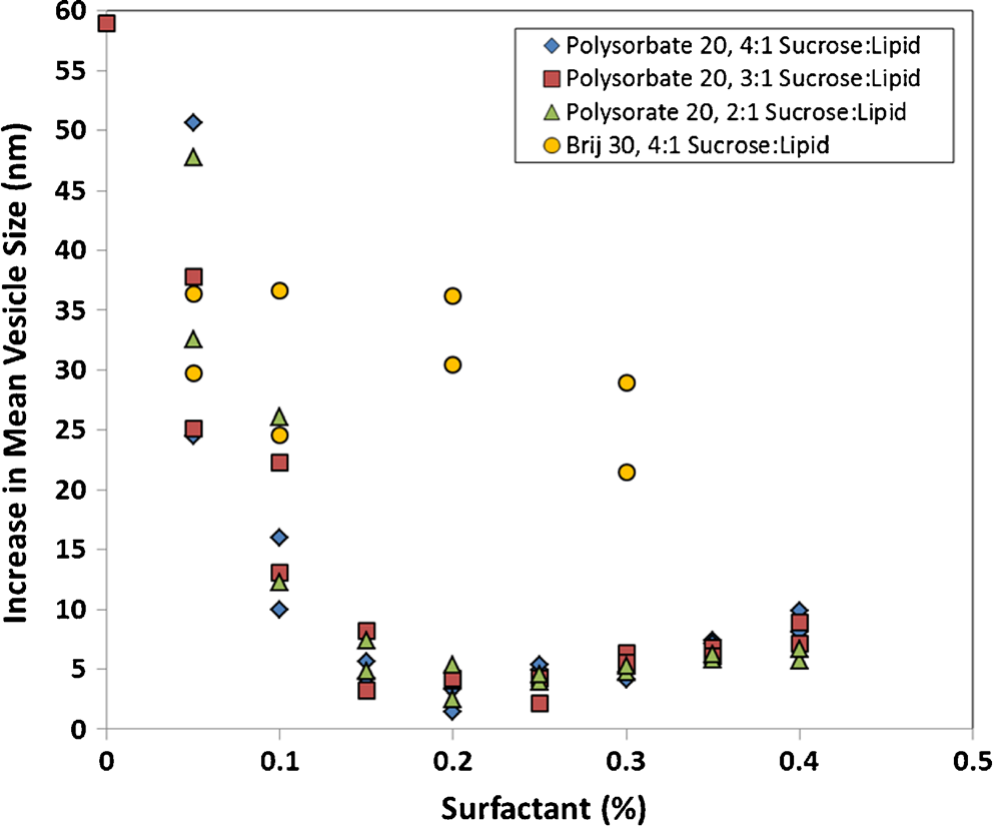
The increase in the mean vesicle size after freeze-thaw for the 12.5 mg/ml CFI formulations containing polysorbate 20 and either 45, 67.5, or 90 mg/ml sucrose (representing 2:1, 3:1, or 4:1 sucrose to lipid (w/w)) or Brij 30 and 90 mg/mL sucrose (representing 4:1 sucrose to lipid (w/w)). The increase in mean vesicle size after freeze-thaw is calculated from the data reported in Table I.

The mean vesicle size before and after freeze-thaw for the 12.5 mg/ml CFI formulations containing 2:1, 3:1, or 4:1 sucrose to lipid, and 0.05 to 0.4% polysorbate 20, is reported in duplicate in Table I. The addition of polysorbate 20 to the CFI formulation containing sucrose resulted in a minimal increase (up to 4 nm) in the mean vesicle size compared to the control CFI formulation without polysorbate 20 or sucrose (91.0 nm). This result is consistent with a previous report of a small increase in vesicle size upon addition of polysorbate 20 to the CFI formulation without sucrose (11). After freeze-thaw, there was a significant increase in mean vesicle size of 25 to 50 nm for the liposomal ciprofloxacin samples containing the least amount of polysorbate 20 (0.05%, Fig. 1) but this is less than the 59 nm vesicle size increase observed for CFI in the absence of polysorbate 20 (Table I). Higher concentrations of polysorbate 20 resulted in even smaller increases in vesicle size (Fig. 1). For example, there was an increase of only 2 to 8 nm in mean vesicle size for the CFI samples containing 0.15 to 0.35% polysorbate 20 (Fig. 1). Thus, the presence of surfactant impacts the final vesicle size following freeze-thaw. The higher surfactant concentrations may reduce the pool of drug that is available within the vesicles to form nanocrystals and thus influence the ultimate size of the vesicle following nanocrystal formation and growth.

**Table I:** The Vesicle Size Distribution Before and After Freeze-Thaw At −50°C of the 12.5 mg/ml CFI Formulations, Containing 45, 67.5, or 90 mg/ml Sucrose (representing 2:1, 3:1, or 4:1 sucrose to lipid (w/w)) and Varying Amounts of Polysorbate 20 or Brij 30

Surfactant (%)	2:1 Sucrose:Lipid (w/w)		3:1 Sucrose:Lipid (w/w)		4:1 Sucrose:Lipid (w/w)	
	Before	After	Before	After	Before	After
Polysorbate 20						
0	ND		91.0 [25.7]	150.0 [75.5]	ND	
0.05	91.5 [25.3]	124.1 [53.5]	90.9 [27.2]	116.0 [43.5]	91.4 [25.3]	115.9 [43.0]
		139.3 [67.5]		138.7 [72.0]		142.0 [68.8]
0.10	91.4 [27.3]	103.7 [34.4]	91.1 [23.9]	104.2 [32.7]	92.0 [16.6]	102.0 [34.0]
		117.5 [41.4]		113.4 [40.8]		108.0 [39.5]
0.15	92.3 [29.0]	97.1 [32.3]	91.8 [24.5]	95.0 [32.6]	92.4 [23.5]	96.6 [27.1]
		99.7 [36.7]		100.0 [32.3]		98.0 [27.8]
0.20	92.8 [22.3]	95.3 [34.7]	91.9 [28.0]	95.9 [31.2]	93.7 [23.7]	97.0 [34.5]
		98.2 [29.8]		96.1 [34.2]		95.2 [33.0]
0.25	93.0 [25.4]	96.9 [29.6]	93.8 [22.8]	98.0 [28.4]	93.0 [24.7]	97.9 [22.6]
		97.6 [33.6]		96.0 [33.4]		98.4 [28.8]
0.30	93.7 [25.2]	98.4 [28.7]	93.7 [22.0]	100.0 [29.2]	95.4 [20.3]	99.5 [30.3]
		99.0 [27.9]		99.2 [18.0]		100.1 [29.7]
0.35	93.9 [25.0]	99.7 [27.4]	94.1 [25.4]	100.9 [32.0]	93.3 [18.2]	100.7 [33.0]
		100.1 [32.6]		100.2 [32.0]		100.4 [34.3]
0.40	93.9 [23.1]	99.6 [34.2]	94.4 [25.9]	101.5 [36.0]	95.3 [28.9]	103.4 [29.0]
		100.6 [38.3]		103.3 [33.1]		105.2 [27.7]
Brij 30						
0.05	ND				92.1 [22.7]	121.8 [45.0]
						128.5 [53.0]
0.10					93.7 [23.4]	118.3 [35.2]
						130.3 [60.3]
0.20					94.4 [22.4]	124.8 [43.2]
						130.6 [64.3]
0.30					96.5 [23.3]	118.0 [44.9]
						125.4 [58.5]

Vesicle size data are reported as the mean (in nm) and [SD]. Duplicate freeze-thaw experiments were performed to characterize the repeatability of the effect of freeze-thaw on the size of the liposomes. *ND* Not Done

#### CFI Containing Brij 30

The mean vesicle size, before and after freeze-thaw, for the 12.5 mg/ml CFI formulations containing 0.05, 0.1, 0.2, and 0.3% Brij 30 were only performed using the highest cryoprotectant concentration of 90 mg/ml sucrose (Table I). Similar to what was observed for CFI containing polysorbate 20, there was an increase in mean vesicle size after freeze-thaw. However, while the presence of moderate amounts of polysorbate 20 (*e.g.*, 0.15 to 0.25%) resulted in very small increases in mean vesicle size of 3 to 5 nm, for Brij 30 there was a large increase in mean vesicle size (of 20 to 40 nm) across the entire range of 0.05 to 0.3% Brij 30, suggesting a different response to freeze-thaw than for the CFI samples containing polysorbate 20 (Fig. 1).

### Drug Encapsulation Before and After Freeze-Thaw

#### CFI Containing Polysorbate 20

**Fig. 2:**
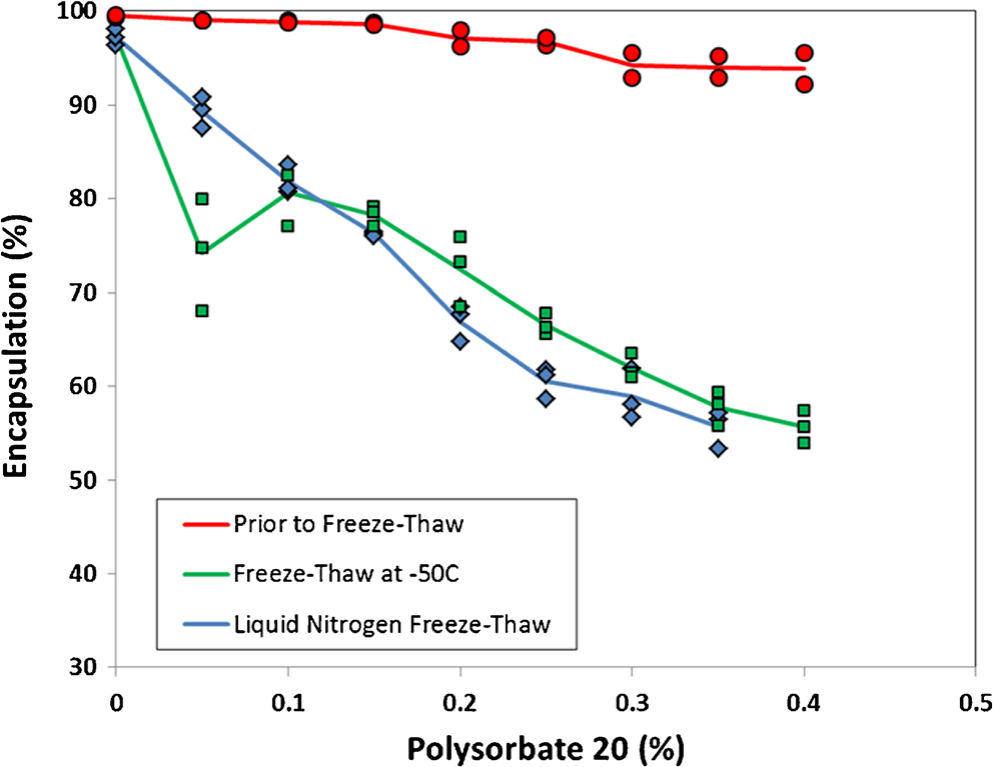
The effect of addition of polysorbate 20 on the state of ciprofloxacin encapsulation for 12.5 mg/ml CFI containing 90 mg/mL sucrose (4:1 sucrose to lipid (w/w)) before and after freeze-thaw in either liquid nitrogen or at −50°C. CFI at 50 mg/ml ciprofloxacin was diluted to a final concentration of ~12.5 mg/ml with sucrose (180 mg/ml), water, and polysorbate 20 (1 or 10%) to achieve a final surfactant concentration of 0.05, 0.1, 0.15, 0.2, 0.25, 0.3, 0.35 or 0.4%. After vortexing and allowing each sample to equilibrate for at least 30 min, the ciprofloxacin encapsulation state was determined by centrifugal filtration. Red circles are prior to freeze-thaw, green squares are after freeze-thaw at −50°C and blue diamonds are after freeze-thaw in liquid nitrogen. Each data point is a single measurement and the lines represent the average.

Previously it was shown that the addition of polysorbate 20 (0.2 to 0.4%) to liposomal ciprofloxacin vesicles in a hyperosmotic environment caused large losses in encapsulated drug of between 20 and 40% due to osmotic swelling of the liposomes during association of the surfactant with the liposome membrane (11). In contrast, in these studies the presence of sucrose in the external buffer was expected to limit the amount of encapsulated drug loss upon addition of surfactant to the 12.5 mg/ml CFI, and that was indeed the case. Only a small loss in drug encapsulation (<10%) resulted from the addition of up to 0.4% polysorbate 20 to 12.5 mg/mL CFI containing 90 mg/mL sucrose (Fig. 2). Similar encapsulation profiles were also observed for the CFI formulations containing 45 or 60 mg/mL sucrose upon addition of polysorbate 20 (data not shown).

After freeze-thaw at −50°C or in liquid nitrogen, increasing concentrations of surfactant led to greater loss in drug encapsulation, indicating release of encapsulated drug in response to the stress of freeze-thaw (Fig. 2). Samples containing the most polysorbate 20 (0.35 to 0.4%) had the greatest decrease in drug encapsulation to ~55%, *via* either freezing method (Fig. 2). There was general agreement with the two freezing methods except for CFI samples containing the lowest concentration of surfactant, 0.05% polysorbate 20. For that sample, freezing in liquid nitrogen produced results in line with the general trend whereas freezing at −50°C resulted in more variable and greater losses in drug encapsulation (Fig. 2).

#### CFI Containing Brij 30

**Fig. 3:**
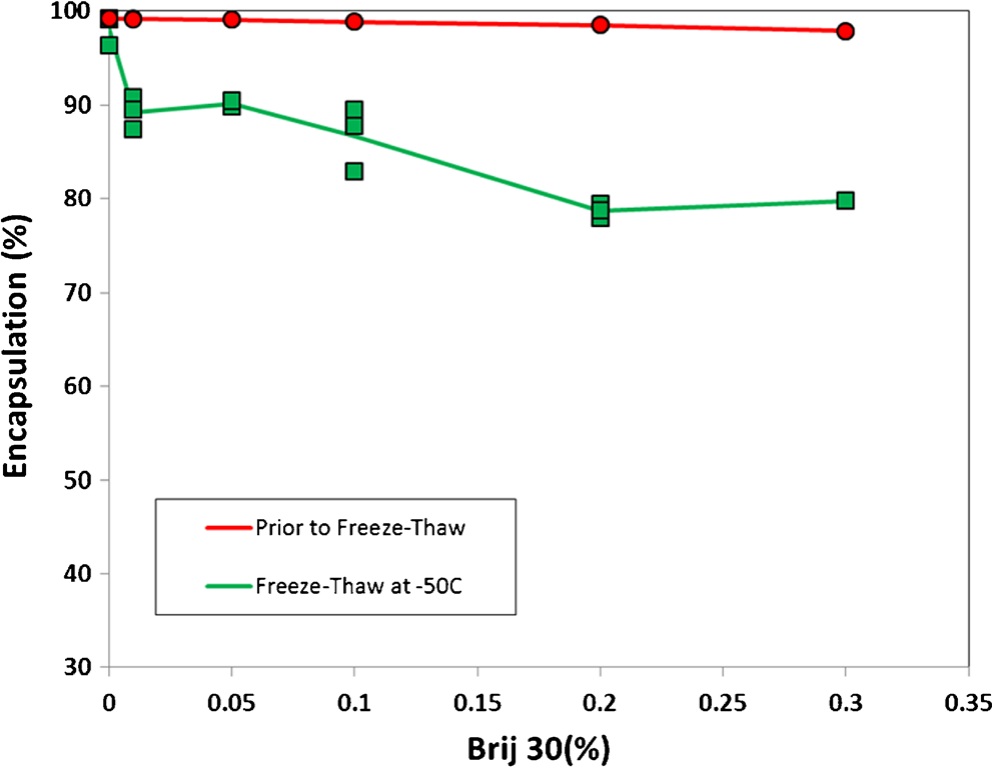
The effect of addition of Brij 30 on the state of ciprofloxacin encapsulation for 12.5 mg/ml CFI containing 90 mg/mL sucrose (4:1 sucrose to lipid (w/w)) before and after freeze-thaw at −50°C. CFI at 50 mg/ml ciprofloxacin was diluted to a final concentration of ~12.5 mg/ml with sucrose (180 mg/ml), water, and Brij 30 (1 or 10%) to achieve a final surfactant concentration of 0.01, 0.05, 0.1, 0.2, and 0.3%. After vortexing and allowing each sample to equilibrate for at least 30 min, the ciprofloxacin encapsulation state was determined by centrifugal filtration. Red circles are prior to freeze-thaw and green squares are after freeze-thaw at −50°C. Each data point represents a single measurement and the lines represent the average.

Prior to freeze-thaw the Brij 30 surfactant did not cause meaningful changes in drug encapsulation with less than 3% drug loss even for CFI samples containing the highest amount (0.3%) of Brij 30 (Fig. 3). After freeze-thaw, there was a significant loss in encapsulated drug ranging up to ~20% for CFI containing 0.3% Brij 30 surfactant (Fig. 3). Compared to polysorbate 20, Brij 30 appeared to be less destabilizing to the liposomal ciprofloxacin vesicles during the freeze-thaw process resulting in a greater retention of encapsulated drug.

### Cryo-TEM Imaging to Evaluate the Kinetics and Morphology of Nanocrystal Formation

#### CFI Containing Polysorbate 20

**Fig. 4:**
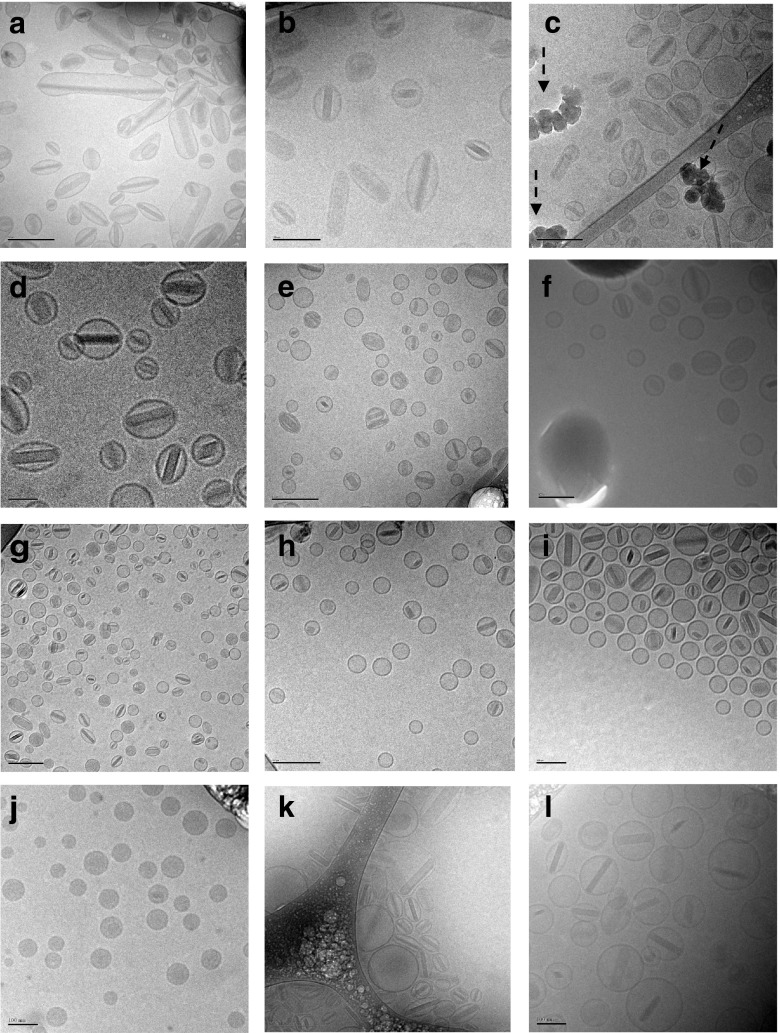
Cryo-TEM micrographs of 12.5 mg/ml CFI, 90 mg/ml sucrose, pH 6.0 liposomes after freeze-thaw in liquid nitrogen. The scale bar in the bottom left-hand corner of micrographs A, C, E, G, H and K is 200 nm, in micrographs B, F, I, J and L is 100 nm and in micrograph D is 50 nm. (**a**) 0.05% polysorbate 20, 45 min post freeze-thaw; (**b**) 0.05% polysorbate 20, 140 min post freeze-thaw; (**c**) 0.05% polysorbate 20, 16.7 h post freeze-thaw; (**d**) 0.1% polysorbate 20, 40 min post freeze-thaw; (**e**) 0.1% polysorbate 20, 140 min post freeze-thaw; (**f**) 0.1% polysorbate 20, 16.7 h post freeze-thaw; (**g**) 0.2% polysorbate 20, <1 min post freeze-thaw; (**h**) 0.2% polysorbate 20, 120 min post freeze-thaw; (**i**) 0.2% polysorbate 20, 16.3 h post freeze-thaw; (**j**) 0.05% Brij 30, prior to freeze-thaw; (**k**) 0.05% Brij 30 < 30 min after freeze-thaw; (**l**) 0.25% Brij 30 < 30 min after freeze-thaw. The dashed arrows in C show ice artifacts introduced during sample preparation. All samples were applied at a concentration of ~10 mg/ml liposomes.

Cryo-TEM imaging was undertaken to determine if the formation of nanocrystals within the vesicles occurred post freeze-thaw in liquid nitrogen for the CFI formulations containing surfactant as was previously reported for the CFI formulations without surfactant, and if so, the effect of surfactant concentration on crystal structure (12). In order to understand the kinetics and stability of the nanocrystal formation process, 12.5 mg/ml CFI samples containing surfactant were analyzed by cryo-TEM immediately after thawing and then at various time points up to 17 h post freeze-thaw in liquid nitrogen. To characterize the effect of different surfactant levels on the structures of the nanocrystals and the liposome morphology, CFI samples contained 4:1 (w/w) sucrose to lipid and either 0.05, 0.1 or 0.2% polysorbate 20. The presence of nanocrystals was observed in every sample, even immediately after thawing (Fig. 4a, d and g), indicating that the formation of nanocrystals was very rapid. For all conditions, the liposomes retained their unilamellar structure.

Similar to the cryo-TEM imaging of nanocrystalline CFI in the absence of surfactant (12), in the presence of only 0.05% polysorbate 20 the liposomes were elongated with the vast majority of liposomes showing the presence of internalized nanocrystalline structures (Fig. 4a). After up to 17 h post freeze-thaw, there did not appear to be a qualitative change in the cryo-TEM images with the vesicles remaining elongated and almost all of them continuing to show the presence of nanocrystalline structures (Fig. 4b–c).

For the liposomes containing 0.1% polysorbate 20, the nanocrystalline structures also formed immediately, but the liposomes remained fairly circular in appearance (Fig. 4d) comparable in size to the unfrozen CFI control images (12, 15). There was a small minority of liposomes without any nanocrystals, and these were predominantly of lighter density than for the control unfrozen CFI images but of comparable density to the empty liposomes (12), suggesting that they no longer contained a significant amount of drug. The proportion of ‘empty’ vesicles did not appear to change over time for the CFI samples containing 4:1 (w/w) sucrose to lipid whereas for the CFI samples containing 3:1 (w/w) sucrose to lipid the proportion of ‘empty’ liposomes appeared to increase over the 17 h time period (data not shown).

For the CFI samples containing 0.2% polysorbate 20, the observations were similar to those described for the CFI samples containing 0.1% polysorbate 20, except that there were an even greater proportion of empty liposomes initially and that proportion increased over the 17 h time period post freeze-thaw. While it is not possibly to strictly quantify the proportion of empty liposomes *versus* those containing nanocrystals by cryo-TEM imaging due to varying film thickness and sorting mechanisms in the cryo-TEM sample matrix (22), the population of empty liposomes was estimated to be ~40% of the total liposomes in the cryo-TEM images for the CFI samples containing 0.2% polysorbate 20 after freeze-thaw.

#### CFI Containing Brij 30

CFI samples at 12.5 mg/ml containing 90 mg/ml sucrose and either 0.05 or 0.25% Brij 30 were analyzed by cryo-TEM imaging before and after freeze-thaw in liquid nitrogen to observe the effect on vesicle morphology and the existence of nanocrystals. These two surfactant concentration levels were chosen to bracket both the low and high end of the surfactant range. The cryo-TEM images of the control CFI formulation containing 0.05% Brij 30 (Fig. 4j) or 0.25% Brij 30 (data not shown) were comparable to that for the control CFI without surfactant (12), displaying unilamellar liposomes around 80–90 nm in size. After freeze-thaw, nanocrystals were observed in both samples containing Brij 30 (Fig. 4k and l), similar to what was observed for the studies with polysorbate 20; however, the liposomes generally remained unilamellar in structure. For the liposomes containing 0.05% Brij 30, the nanocrystalline structures were observed within elongated liposomes (Fig. 4k), not unlike what was observed in the studies with 0.05% polysorbate 20. In contrast, using 0.25% Brij 30, while the liposomes were more circular in shape, they were larger than observed in the studies with 0.1 to 0.2% polysorbate 20, consistent with the differences observed in vesicle size by DLS (Table I). More empty liposomes were also observed in the cryo-TEM images for the higher Brij 30 concentration (Fig. 4l*versus* k), similar to what was observed for polysorbate 20 (Fig. 4g*versus* d).

### Effect on IVR Profile

#### CFI Containing Polysorbate 20

**Fig. 5:**
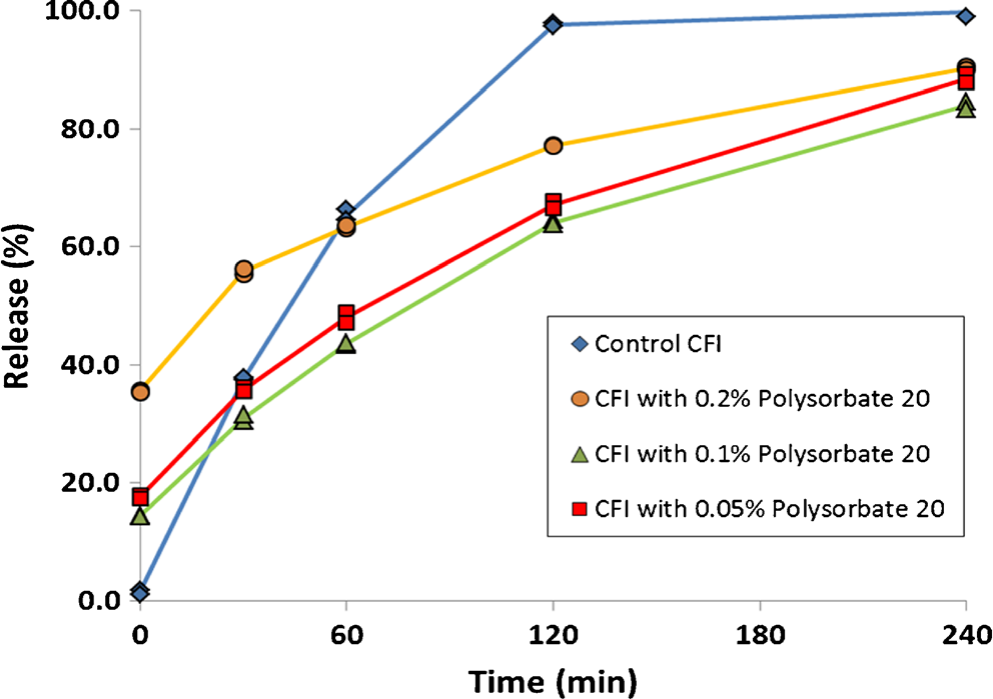
Evaluation of the effect of freeze-thaw at −50°C on the IVR profiles of CFI formulations at pH 6.0 containing 90 mg/ml sucrose and polysorbate 20 after freeze-thaw. The CFI formulations (at 12.5 mg/ml) were diluted to 50 μg/ml ciprofloxacin in HEPES buffered saline (HBS) prior to a 1:1 dilution in bovine serum to measure the release of ciprofloxacin after incubation at 37°C for up to 4 h. IVR profiles are shown for the CFI control (no freeze-thaw, blue diamonds), CFI containing 0.05% polysorbate 20 after freeze-thaw (red squares), CFI containing 0.1% polysorbate 20 after freeze-thaw (green triangles), and CFI containing 0.2% polysorbate 20 after freeze-thaw (yellow circles). Duplicate samples were analyzed at each time point.

To determine how the presence of the nanocrystals affected the drug release rate, the IVR profiles of the 12.5 mg/ml CFI formulation containing 90 mg/ml sucrose and either 0.05, 0.1 or 0.2% polysorbate 20, pH 6.0 were obtained after freeze-thaw at −50°C and compared to that for the control CFI which was not frozen (Fig. 5). The IVR profiles after freeze-thaw for all three CFI formulations containing surfactant demonstrated slower initial release rates compared to the unfrozen control, consistent with the presence of drug nanocrystals within the vesicles. There was an initial “burst” of 17, 14 and 35% drug at T = 0 for the formulations containing 0.05, 0.1 and 0.2% polysorbate 20, respectively, generally consistent with the trend in loss of encapsulated drug reported earlier after freeze-thaw at −50°C (Fig. 2). None of the IVR profiles for the experimental samples achieved complete release of drug over the 240 min period, but achieved 83 to 89% release by the last time point in the assay.

By visual inspection, there appears to be a meaningful difference in the release profiles for all three polysorbate 20-containing CFI formulations after freeze-thaw compared to the control CFI formulation (without nanocrystals). Similarity factor (f_2_) analysis was used to quantify the difference between the profiles, with an f_2_ value below 50 indicating a significant difference (Table II). Post freeze-thaw, the similarity factor analysis confirmed that all three CFI formulations containing polysorbate 20 after freeze-thaw had distinct IVR profiles compared to the unfrozen control formulation, with similarity factors less than 50: 37.2, 34.6 and 34.9 for the formulations containing 0.05, 0.1 and 0.2% polysorbate 20, respectively (Table II). The only profiles found to be similar to each other by similarity factor analysis were for those containing 0.05 and 0.1% polysorbate 20 after freeze-thaw.

**Table II:**
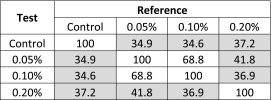
Similarity Factor Analysis (f_2_) for CFI Formulations Containing Various Amounts of Polysorbate 20 After Freeze-Thaw at −50°C *Versus* the CFI Control

Shaded cells indicate lack of similarity

The addition of polysorbate 20 to CFI causes both an increase in non-encapsulated drug and a change in nanocrystalline character after freeze-thaw; *i.e.*, shorter crystals within more circular vesicles. To better understand the influence of these changes in the nanocrystalline structure on release rate, another freeze-thaw study was performed this time including the CFI formulation without polysorbate 20 and using liquid nitrogen to freeze the samples, to mimic the conditions used for cryo-TEM analysis.

**Fig. 6:**
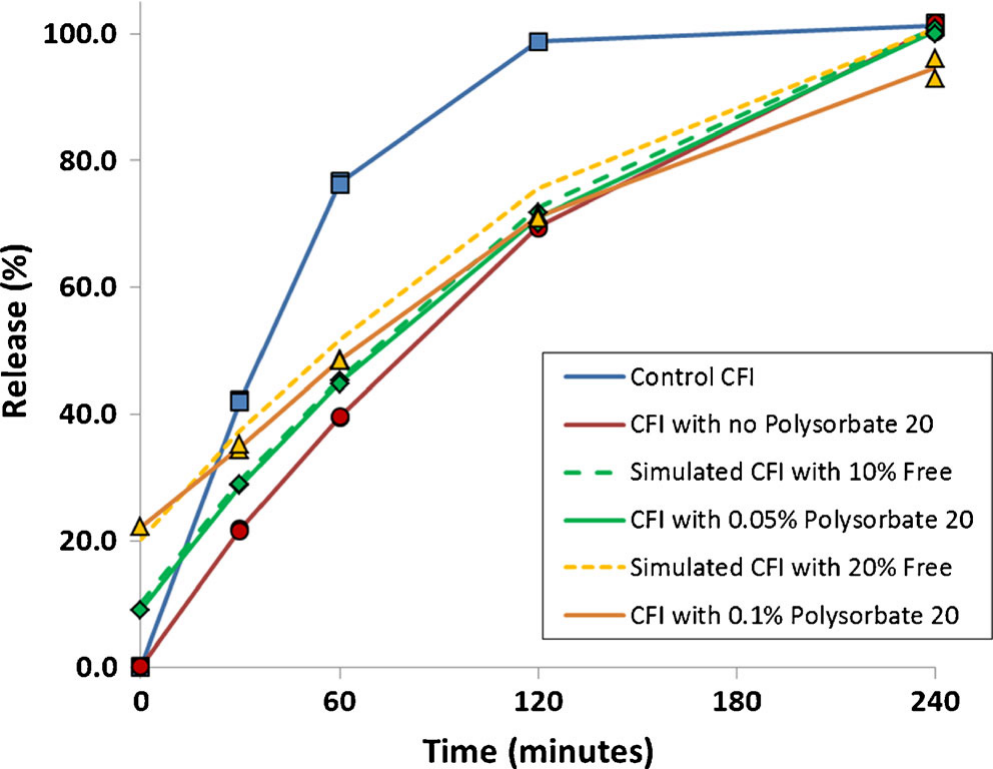
Evaluation of the effect of freeze-thaw in liquid nitrogen on the IVR profiles of CFI formulations at pH 6.0 containing 90 mg/ml sucrose and 0, 0.05 or 0.1% polysorbate 20 after freeze-thaw. The CFI formulations (at 12.5 mg/ml) were diluted to 50 μg/ml ciprofloxacin in HEPES buffered saline (HBS) prior to a 1:1 dilution in bovine serum to measure the release of ciprofloxacin after incubation at 37°C for up to 4 h. IVR profiles are shown for the CFI control prior to freeze-thaw (*blue diamonds*) or after freeze-thaw (*red circles*), CFI containing 0.05% polysorbate 20 after freeze-thaw (*green diamonds*), CFI containing 0.1% polysorbate 20 after freeze-thaw (*yellow triangles*). Duplicate samples were analyzed at each time point. Two simulated curves were generated by adjusting the release from the CFI sample without polysorbate 20 after freeze-thaw by assuming the addition of either 10% free drug (*green dashed line*) or 20% free drug (*yellow dashed line*) and normalizing the total drug release to 100%.

The IVR profiles after freeze-thaw for the CFI formulations with or without polysorbate 20 again demonstrated slower initial release rates compared to the unfrozen control (Fig. 6), similar to the results shown previously in Fig. 5. The nanocrystalline profiles also demonstrate a statistically significant difference to the unfrozen control CFI using similarity factor analysis (Table III). The amount of unencapsulated drug initially was 0.2, 9.1 and 22.2% for the formulations containing none, 0.05, and 0.1% polysorbate 20, respectively, consistent with the trend in loss of encapsulated drug reported earlier after liquid nitrogen freeze-thaw (Fig. 2). To compare just the contribution of the encapsulated drug to the rate of release, the normalized initial rate of release was calculated. The normalized initial rate of release was 42, 22, 22 and 16%, respectively, for the CFI control and the three CFI formulations containing none, 0.05 and 1% polysorbate 20 after freeze-thaw. The normalized initial rate of release for all three nanocrystalline formulations was about half that of the CFI control. Thus, while the three CFI formulations have different nanocrystalline structures (Fig. 4 and (12)), there is little change to the initial rate of drug release compared to each other.

**Table III:**
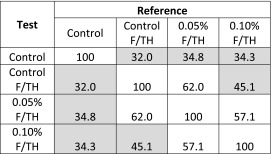
Similarity Factor Analysis (f_2_) for CFI Formulations Containing Various Amounts of Polysorbate 20 After Freeze-Thaw in Liquid Nitrogen *Versus* the CFI Control

Shaded cells indicate lack of similarity

Another way to compare the release profiles from the encapsulated drug alone is to take the release profile for the nanocrystalline formulation without polysorbate 20 and simulate the addition of 10 or 20% unencapsulated drug to the profile (to match the T_0min_ for the two formulations containing polysorbate 20). The contribution from the nanocrystals is also reduced by 10 or 20%, respectively, in this simulation so that the total drug release still represents 100%. These profiles are shown by the yellow and green dashed lines in Fig. 6. The simulated profile for the nanocrystalline formulation with 10% unencapsulated drug very closely matches that for the CFI formulation containing 0.05% polysorbate 20 with a similarity factor (f_2_) of 92.3, very near 100. The simulated profile for the nanocrystalline formulation with 20% unencapsulated drug is not statistically different from that for the CFI formulation containing 0.1% polysorbate 20 with a similarity factor (f_2_) of 69.2, exceeding 50. Discounting the presence of the initial unencapsulated drug, these results confirm that the rate of encapsulated drug release from the three nanocrystalline liposomal formulations is not statistically different. This result also suggests that the permeability of the lipid membrane was unaffected by the presence of 0.05 or 0.1% polysorbate 20, and is consistent with previous observations (11).

#### CFI Containing Brij 30

**Fig. 7:**
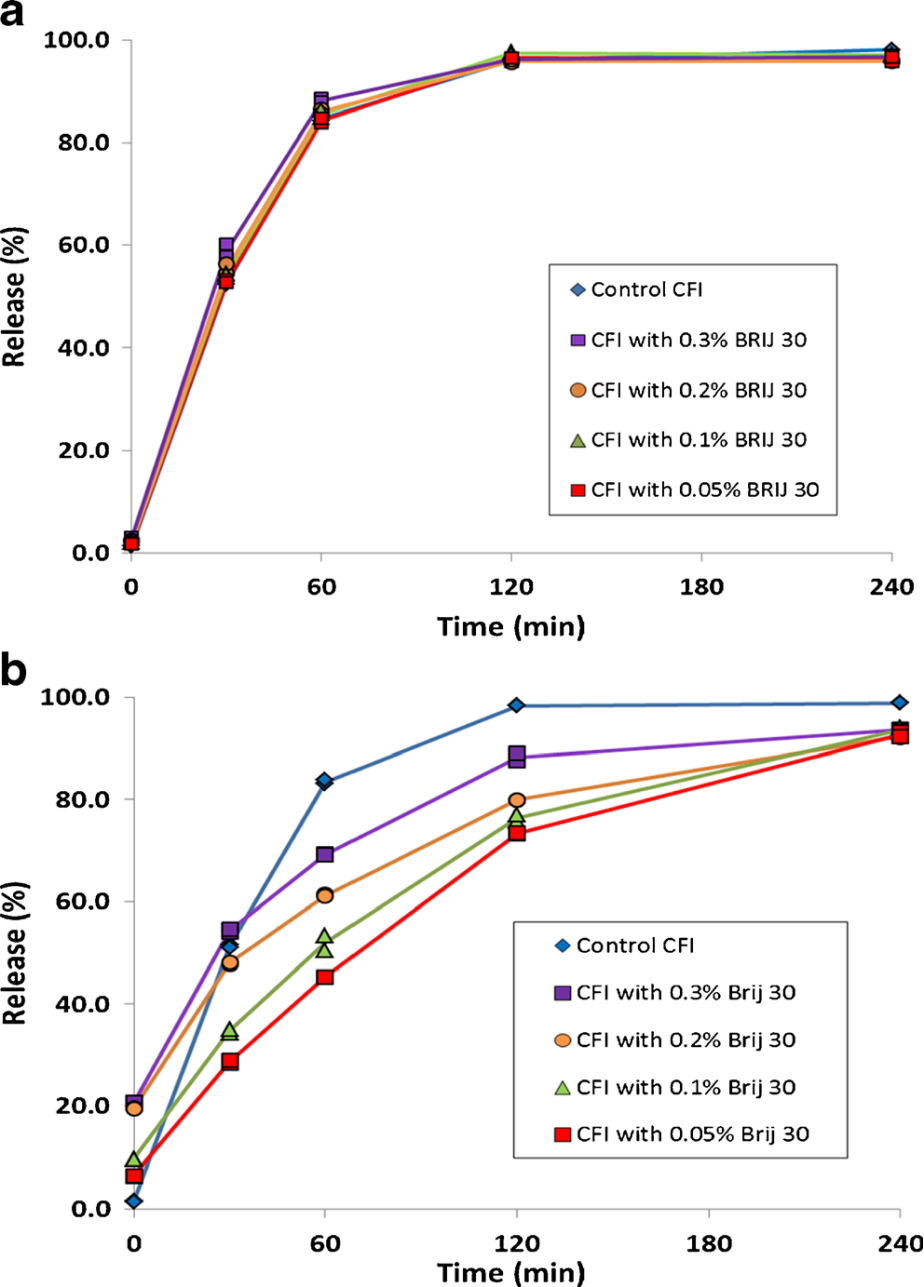
Evaluation of the effect of freeze-thaw at −50°C on the IVR profiles of CFI formulations at pH 6.0 containing 90 mg/ml sucrose and Brij 30 before (**a**) and after (**b**) freeze-thaw. The 12.5 mg/ml CFI formulations were diluted to 50 μg/ml ciprofloxacin in HEPES buffered saline (HBS) prior to a 1:1 dilution in bovine serum to measure the release of ciprofloxacin after incubation at 37°C for up to 4 h. IVR profiles are shown for the CFI control (no freeze thaw, *blue diamonds*), CFI containing 0.05% Brij 30 (*red squares*), CFI containing 0.1% Brij 30 (*green triangles*), CFI containing 0.2% Brij 30 (*orange circles*), and CFI containing 0.3% Brij 30 (*purple squares*). Duplicate samples were analyzed at each time point.

Prior to freeze-thaw, the presence of 0.05% Brij 30 in the CFI formulation had a negligible effect on the IVR profile compared to the control CFI sample (Fig. 7a). The samples containing 0.1, 0.2, or 0.3% Brij 30 had slightly faster initial release rates at the 30 min time point (Fig. 7a), with the fastest initial release rate for the CFI sample with the highest surfactant concentration. The addition of Brij 30 surfactant to liposomal ciprofloxacin does not result in a significant change in the IVR profile when evaluated by similarity factor analysis (Table IV). All of the similarity factor values exceed 72, above the key value of 50, indicating that there is not a meaningful difference between any of the IVR profiles for the various samples prior to freeze-thaw.

**Table IV:** Similarity Factor Analysis (f_2_) for CFI Formulations Containing Various Amounts of Brij 30 Before Freeze-Thaw *Versus* the CFI Control

Test	Reference				
	Control	0.05%	0.10%	0.20%	0.30%
Control	100	93.4	90.3	84.6	72.7
0.05%	93.4	100	92.2	88.3	73.5
0.10%	90.3	92.2	100	91.8	78.7
0.20%	84.6	88.3	91.8	100	83.4
0.30%	72.7	73.5	78.7	83.4	100

After freeze-thaw, all CFI samples containing Brij 30 showed an increase in non-encapsulated drug at the T = 0 min time point in the IVR assay, ranging from 6 to 21% (Fig. 7b), not too dissimilar from the drug encapsulation values reported in Fig. 3. This initial “burst” was followed by a slower initial release profile compared to that for the unfrozen control CFI sample, as would be expected if drug nanocrystals had formed as a result of freeze-thaw (Fig. 7b). All samples approached complete drug release by the last time point in the IVR assay. After freeze-thaw, all of the nanocrystalline-containing Brij 30 formulations had a significant difference in the IVR profiles compared to that of the control liposomal ciprofloxacin formulation; the similarity factors range from 31.9 to 46.1 (Table V). The normalized initial rate of release was 51, 22, 25, 28 and 34% for the CFI control and the four CFI formulations containing 0.05, 0.1, 0.2 and 0.3% Brij 30, respectively, after freeze-thaw. The normalized initial rate of release for all three nanocrystalline formulations was about half that of the CFI control.

**Table V:**
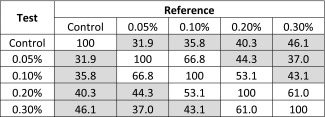
Similarity Factor Analysis (f_2_) for CFI Formulations Containing Various Amounts of Brij 30 After Freeze-Thaw *Versus* the CFI Control

Shaded cells indicate lack of similarity

## Discussion

The application of nanotechnology approaches for treatment of lung disease is an emerging area that has recently been reviewed (23, 24). The ability to customize release rates of these nanotechnology formulations by simple operations and without any major qualitative or quantitative changes in the composition of the drug product might be an attractive path for personalized medicine (25). For example, the nature of chronic lung infections varies even in the same individual over time, and different individuals with the same disease may have different strains of the same bacteria or a different set of co-infections. Furthermore, the host immune system reacts to the nature of the lung microbiome impacting the progression of the disease (26). To get the optimum therapeutic response – the balance of efficacy and safety – a more sophisticated approach to re-establish homeostasis in each individual patient may be preferable to the ‘one size fits all’ therapeutic approach that is often necessitated by the nature of the current pharmaceutical dosage forms and the regulatory approval process for those medications.

With respect specifically to the example in this research, liposomal ciprofloxacin is being developed as an inhaled therapy to treat lung infections (4–6). While the release profile of the current liposomal ciprofloxacin product in development, Pulmaquin, appears to be appropriate for non-CF bronchiectasis patients colonized with *P. aeruginosa* (4–6), there may be other lung infections in this or other patient populations for which a ‘fine tuning’ of ciprofloxacin release would be desirable. For example, a higher early spike of ciprofloxacin may be warranted to kill certain types of bacteria present in the lungs; *e.g.*, those with higher MIC values, and this could be achieved by addition of polysorbate 20 or Brij 30 to the formulation to increase the “burst” of antibiotic. For patients with intracellular infections in their alveolar macrophages; *e.g.*, non TB mycobacteria, it may be desirable to delay release until the macrophages have time to take up the liposomes. A liposomal formulation containing drug nanocrystals thus might be more efficacious for this patient population (12). A simple freeze-thaw procedure to transform the drug into nanocrystalline form prior to inhaled administration could accomplish this goal (12). Other infectious states might ideally be treated by a combination of both a high initial bolus and a slower release profile which could be achieved by addition of surfactant to the liposomal ciprofloxacin formulation prior to freeze-thaw. In summary, we have described a number of methodologies to tailor a liposomal formulation’s release profile to personalize treatment.

It was previously shown that liposomal encapsulated ciprofloxacin could be converted into nanocrystalline form following freeze-thaw; the liposomes retained structural integrity to freeze-thaw by the addition of a cryoprotectant, either sucrose or trehalose (12). The addition of cryoprotectant is known to limit mechanical damage to liposomes by ice crystals which can cause vesicle rupture during freezing or thawing (27–29). The current studies extend the previous results by investigating whether surfactant interactions could be used to alter the solid state behavior of the encapsulated drug and thus further modulate the drug release profile. In these studies the formulations with the best stability and least loss of encapsulated drug after freeze-thaw contained a 4:1 (w/w) ratio of sucrose to lipid. A ratio of 2:1 (w/w) sucrose to lipid provided some protective benefit but generally resulted in greater loss in encapsulated drug than for the formulations with higher levels of sucrose (data not shown).

The mechanisms underlying the effect of the surfactants on the release rate are not yet fully understood but it is possible to form a coherent qualitative picture. In the previous studies without surfactant it was generally observed that each liposome contained only one nanocrystal after freeze-thaw (12). The ‘one nanocrystal per liposome’ observation was also evident in the new studies for CFI formulations containing low levels of polysorbate 20 (*i.e.*, 0.05%) immediately after freeze-thaw (Fig. 4a) and these liposomes retained their encapsulated nanocrystal structures for at least 17 h post freeze-thaw (Fig. 4c). The ‘one nanocrystal per liposome’ was also observed for CFI formulations containing low concentrations of Brij 30 (*i.e.*, 0.05%) immediately after freeze-thaw (Fig. 4k). Together, these observations suggest that the rate of crystal growth within each liposome is very fast, occurring within seconds of thawing. Another observation is that the amount of unencapsulated drug generally increased after freeze-thaw with increasing surfactant concentration for both polysorbate 20 (Fig. 2) and Brij 30 (Fig. 3) compared to the CFI samples without surfactant which had negligible drug loss after freeze-thaw (Figs. 2 and 3).

**Fig. 8:**
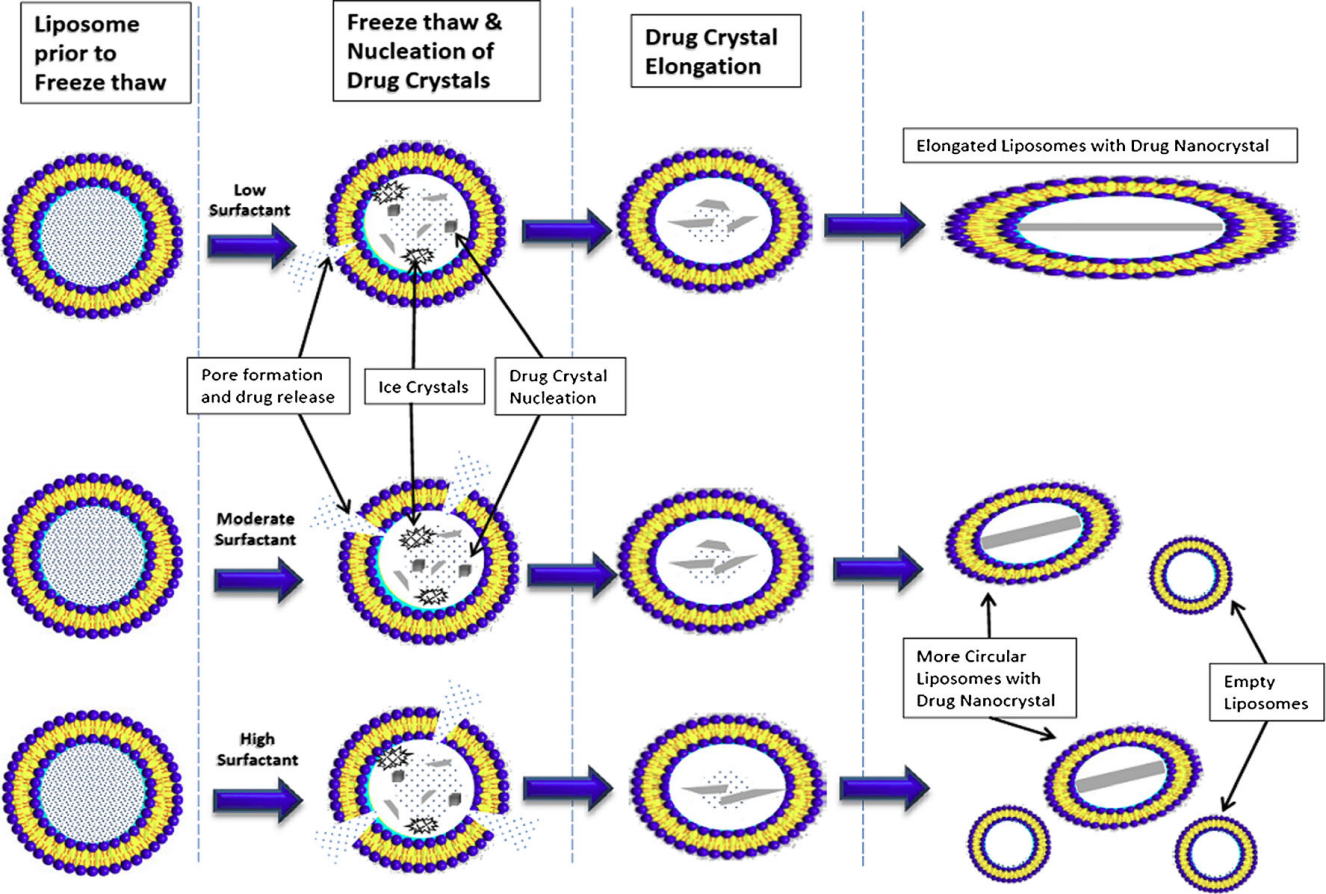
Proposed mechanism for surfactant vesicle interactions during freeze-thaw explain the changes in both vesicle size and drug encapsulation. During freeze-thaw, ice crystals serve as nucleation sites for drug crystal formation. The presence of surfactant causes pore formation with release of drug during the freezing or thawing steps. Higher concentrations of surfactant result in greater overall loss of drug and an increase in the population of empty liposomes. While there may be multiple crystal nucleation events in each liposome, one crystal grows larger at the expense of the others resulting in only one drug nanocrystal per liposome. During the drug crystal growth phase, for higher concentrations of polysorbate 20, the liposomes remain fairly circular due to less drug being present within the vesicles leading to shorter nanocrystals. For low concentrations of polysorbate 20, the vesicles become more elongated to accommodate the longer drug crystal. For Brij 30, the liposome vesicles increase in size due to their association with the surfactant and fully accommodate the growing drug crystal without becoming elongated in shape.

Figure 8 provides a tentative explanation of the mechanisms underlying these observations. The release-rate modulating surfactant (either Brij 30 or Polysorbate 20) causes transient pore formation during the freezing and/or thawing steps resulting in loss of encapsulated drug with greater drug loss for higher surfactant concentrations. This is consistent with studies that show that at low surfactant concentrations, below the liposome-solubilizing level, surfactant molecules can cooperatively assemble into structures which form transient pores in liposomes, allowing release of drug prior to rapid restoration of the membrane integrity (30–32). Higher levels of surfactant would result in a greater number of pores being formed and thus a greater loss of encapsulated drug.

The presence of moderate amounts of polysorbate 20 (*e.g.*, 0.1 and 0.2%) resulted in liposomes which retained their circular shape in cryo-TEM images after freeze-thaw (Fig. 4d and g). The shape of the liposome appears to be the result of the growth of the nanocrystal; long nanocrystals cause the liposomes to elongate. Once a crystallization event is initiated, presumably the nanocrystal will continue to grow until the concentration of ciprofloxacin within the vesicle falls below its solubility limit. For CFI containing 0.1 or 0.2% polysorbate 20, the loss of encapsulated drug may result in shorter nanocrystals which don’t perturb the vesicle shape from its initial circular state.

For low surfactant levels (*e.g.*, 0.05% polysorbate 20), little drug may be released during freeze-thaw which may result in nanocrystals growing larger than the original size of the vesicles. This may lead to elongated vesicles (Fig. 4a) or in some cases, vesicle rupture which will increase the amount of non-encapsulated drug. From these studies it is not possible to know how much of the non-encapsulated drug is produced during the freeze-thaw event *versus* in response to the growing nanocrystal. However, it appears that freeze-thaw at −50°C results in a greater loss in encapsulated drug for CFI containing 0.05% polysorbate 20. These studies were not designed to ascertain the cause of the increased loss of encapsulated drug when frozen at −50°C *versus* using liquid nitrogen. In contrast to the situation with 0.05% polysorbate 20, liposomes containing the same concentration of Brij 30 appear to be more deformable and able to accommodate the larger nanocrystals; thus, there may be less vesicle rupture in response to the growth of the nanocrystals and lower levels of released drug for low Brij 30 concentrations (Fig. 3).

An additional difference between the two surfactants was observed when moderate amounts of surfactant (*e.g.*, 0.2% polysorbate 20 and 0.25% Brij 30) were used. For the CFI formulation containing 0.2% polysorbate 20, after freeze-thaw the nanocrystals were tightly constrained within the liposomes but the vesicle size was relatively unchanged. In contrast, in the presence of 0.25% Brij 30 the liposomes appeared to expand to a greater size than was required to accommodate the nanocrystals. This visual evidence suggests that the CFI vesicles containing Brij 30 could more easily accommodate larger nanocrystals without rupturing and thus explains the higher drug encapsulation values for Brij 30.

The differences in the structure of these surfactants may provide an explanation for the observations: Brij 30 and polysorbate 20 both contain a hydrophobic tail of similar length, while the polar head group for Brij 30 is much smaller than that for polysorbate 20. This may explain why the liposome vesicles incorporating the smaller Brij 30 surfactant molecules are able to flexibly accommodate the growing ciprofloxacin crystals while the vesicles incorporating the polysorbate 20 surfactant molecules with their much larger polar head groups are more constrained and may be less able to flex, leading to destabilization and loss of encapsulated drug during nanocrystal growth.

After freeze-thaw, the CFI formulations containing surfactant all had a population of empty vesicles and a population of vesicles containing nanocrystals and the balance between these two states presumably accounted for the changes in the release profiles. Thus, both aspects of the IVR profile from the CFI liposomes can be modified by polysorbate 20 (Figs. 5 and 6) and Brij 30 (Fig. 7), *i.e.*, the “burst” of drug at the initial time as well as the subsequent slower release profile.

The presence of precipitated drug within liposomes has been described previously for vincristine (13), doxorubicin (2, 14), topotecan (2) and vinorelbin (2, 33). In contrast to these studies where the ciprofloxacin formed nanocrystals in response to freeze-thaw, in the other studies the drug formed precipitates during the loading process. It has also been previously noted that precipitated drug within liposome vesicles leads to a slower release and a longer *in vivo* half-life (2, 13). The rationale for the slower release has been the lower soluble intravesicular drug concentration which is the driving force for drug transport across the liposome membrane (2, 13, 14, 33). If the rate of drug dissolution is fast compared to transport across the lipid membrane, then an almost zero order rate of release can be obtained as was demonstrated for doxorubicin (14) and vinorelbin (33).

However, it was hypothesized that “the rate of dissolution of the intravesicular precipitate could further modulate the internal drug concentration and even become the rate limiting step for release” (33). In this liposomal ciprofloxacin system, different nanocrystalline structures were created by varying the surfactant concentration. If drug dissolution were rate limiting, then it would be likely that a change in crystalline character would lead to differences in the rate of drug release. The nanocrystalline structures were shorter and smaller for the CFI samples containing higher polysorbate 20 concentrations, which might be expected to lead to faster rates of drug dissolution. However, the initial rate of release was unchanged when accounting for the unencapsulated drug (Fig. 6). This result suggests that dissolution may not be rate-limiting for the liposomal systems containing nanocrystalline ciprofloxacin.

The association of the surfactant into the liposome membrane may further influence the membrane permeability and release rate. Polysorbate 20 did not appear to modify the mebrane permeability. However, for Brij 30, the liposome vesicles remain circular but become larger, even greater in size than appears to be necessary to accommodate the nanocrystal (Fig. 4l). Increasing concentrations of Brij 30 resulted in faster initial rates of release from the encapsulated component (Fig. 7) which suggests that the membrane permeability has also been modified. The possibility that dissolution may be rate-limiting cannot be entirely discounted: The faster initial rate of release for CFI containing increasing Brij 30 concentrations could be more rapid dissolution from the shorter drug crystals at the higher surfactant concentrations and not a change in the permeability of the bilayer.

The long term safety and tolerability of these liposomal ciprofloxacin formulations containing Brij 30 or polysorbate 20 surfactant, like every inhaled product, must be assessed in preclinical animal toxicology studies prior to entry into clinical trials. However, the presence of liposomes or surfactants in an inhaled product should not be alarming as the lung is bathed in lung surfactant composed of proteins, cholesterol and lipids (34). Furthermore, a number of synthetic or natural “surfactants” are approved for administration to the lungs to treat respiratory distress syndrome including Infasurf®, Exosurf®, Survanta®, Curosurf®, and Surfaxin® (35). Surfactants are also used to formulate drug suspensions for delivery by nebulization. Marketed metered dose inhaler (MDI) products also use surfactants to improve formulation stability and delivery performance; *e.g.*, oleic acid, PVP, lecithin, and sorbitan oleates.

In conclusion, as previously reported (12), freeze-thawing of liposomally encapsulated ciprofloxacin results in the formation of nanocrystals of ciprofloxacin within the liposomal vesicles with an attenuated ciprofloxacin release rate compared to the unfrozen liposomal formulation. Addition of certain types of surfactants such as polysorbate 20 and Brij 30 can be used to modulate the release rates of ciprofloxacin from these nano-structures, primarily by changing the ratio of the immediate release and sustained release component. These simple operations with modest changes to the qualitative and quantitative composition of the formulation enable fine-tuning of the release rates that may be utilized in personalizing medicines for the individual needs of patients. Some of these liposome formulations containing ciprofloxacin nanocrystals are stable to nebulization and so could be inhaled by patients with lung infections (23, 36).

## Conclusions

We previously showed that the physical state of liposomal-encapsulated ciprofloxacin can be converted to nanocrystalline form following freeze-thaw. These drug nanocrystals introduce a dissolution step and a lower intravesicular drug concentration which reduces the concentration gradient for drug release compared to that of the unfrozen control formulation. The size and shape of the nanocrystals can be further adjusted by the addition of a small amount of a surfactant, either 0.05 to 0.20% polysorbate 20 or 0.05 to 0.25% Brij 30, which results in shorter drug nanocrystals within the liposome vesicles and an increase in unencapsulated drug. The addition of surfactant thus allows for further fine-tuning of the release profile by modifying the ratio of the immediate release and sustained release component as well as potentially changing the permeability of the bilayer. These advances have the potential to transform inhaled antiinfective therapy by providing methodologies that allow the doctor or end user to modify the antibiotic release profile to specifically address the needs of the patient.

## ABBREVIATIONS

API: Active pharmaceutical ingredient
CF: Cystic fibrosis
CFI: Ciprofloxacin (liposomal) for inhalation
Fr/Th: Freeze/Thaw
HBS: HEPES buffered saline
HPLC: High pressure liquid chromatography
HSPC: Hydrogenated soy phosphatidylcholine
IVR: *In vitro* release
MIC: Minimum inhibitory concentration
NCFB: Non cystic fibrosis bronchiectasis
SD: Standard deviation
TB: Tuberculosis
TEA: Triethylamine
TEM: Transmission electron microscopy
